# Copper to Zinc Ratio as Disease Biomarker in Neonates with Early-Onset Congenital Infections

**DOI:** 10.3390/nu9040343

**Published:** 2017-03-30

**Authors:** Monika Wisniewska, Malte Cremer, Lennart Wiehe, Niels-Peter Becker, Eddy Rijntjes, Janine Martitz, Kostja Renko, Christoph Bührer, Lutz Schomburg

**Affiliations:** 1 Institute for Experimental Endocrinology, Charité-Universitätsmedizin Berlin, Augustenburger Platz 1, CVK, D-13353 Berlin, Germany; Monika.Wisniewska@charite.de (M.W.); Lennart.Wiehe@charite.de (L.W.); Niels-Peter.Becker@charite.de (N.-P.B.); Eddy.Rijntjes@charite.de (E.R.); Janine.Martitz@charite.de (J.M.); Kostja.Renko@charite.de (K.R.); 2 Department of Neonatology, Charité-Universitätsmedizin Berlin, Augustenburger Platz 1, CVK, D-13353 Berlin, Germany; Malte.Cremer@charite.de (M.C.); christoph.buehrer@charite.de (C.B.)

**Keywords:** ceruloplasmin, preterm, C reactive protein, interleukin-6, micronutrient.

## Abstract

Copper (Cu) and zinc (Zn) are essential trace elements for regular development. Acute infections alter their metabolism, while deficiencies increase infection risks. A prospective observational case-control study was conducted with infected (*n* = 21) and control (*n* = 23) term and preterm newborns. We analyzed trace element concentrations by X-ray fluorescence, and ceruloplasmin (CP) by Western blot. Median concentration of Cu at birth (day 1) was 522.8 [387.1–679.7] μg/L, and Zn was 1642.4 ± 438.1 μg/L. Cu and Zn correlated positively with gestational age in control newborns. Cu increased in infected newborns from day 1 to day 3. CP correlated positively to Cu levels at birth in both groups and on day 3 in the group of infected neonates. The Cu/Zn ratio was relatively high in infected newborns. Interleukin (IL)-6 concentrations on day 1 were unrelated to Cu, Zn, or the Cu/Zn ratio, whereas C-reactive protein (CRP) levels on day 3 correlated positively to the Cu/Zn -ratio at both day 1 and day 3. We conclude that infections affect the trace element homeostasis in newborns: serum Zn is reduced, while Cu and CP are increased. The Cu/Zn ratio combines both alterations, independent of gestational age. It may, thus, constitute a meaningful diagnostic biomarker for early-onset infections.

## 1. Introduction

Copper (Cu) and zinc (Zn) are trace elements essential for life and constitute components of numerous enzymes with high importance for survival and function of eukaryotic cells [1,2,3,4]. Cu and Zn are necessary for cellular metabolism and the antioxidative defense systems [1,2,3,5]. The regular fetal development and growth critically depend on both Cu and Zn, especially during the maturation of the nervous system [2,4,5]. Similarly, the maturing immune system relies on these trace elements [2,3,5,6], especially for antibody production (Cu, Zn), function of neutrophils and monocytes (Cu) [6], the viability, proliferation, and differentiation of cells of both the innate and adaptive immune system (Zn), as well as for the maintenance of the skin and mucosal barriers (Zn) [3,6].

Both a deficiency and an excess of Cu or Zn can cause harm, so the homeostasis of both elements is strictly regulated [1,2,3]. The main source of both micronutrients is dietary intake [1,3]. The absorption from the gastrointestinal tract depends on the nutritional form and the micronutrient status of the subject [4,7]. The cellular homeostasis is controlled by import and export proteins, cytosolic metallochaperones, glutathione, and metallothioneins [1,2,5]. The latter also acts as a dynamic Cu and Zn pool [3,5]. Around 95% of Cu in blood is bound to liver-derived ceruloplasmin (CP), which is used along with serum Cu concentrations as a biomarker of Cu status [4]. Serum Cu and CP concentrations constitute acute phase reactants [5]. Cu is mainly stored in liver, secreted as Cu-CP complex into blood, and an excess can be eliminated by biliary excretion [1,4]. In contrast to Cu, the protein-mediated transport, storage, and regulated excretion of Zn are more complex and less well understood [3,7].

Cu and Zn deficiencies constitute prevalent and under-diagnosed health risks [1,3]. Neonates and especially preterm infants have a notable risk of Cu and Zn deficiency due to their rapid growth and the concomitant increasing requirement for both micronutrients [4]. On the one hand, Cu or Zn deficiencies impair the immune defense and confer a high susceptibility to infectious diseases [3,4,5,6,8]. On the other hand, acute infections cause an increase in serum Cu in the context of an acute phase response [4,9] and a decrease in serum Zn due to a redistribution into liver and other tissues [3,7,10,11,12]. These two responses to inflammation may feed a vicious cycle of impaired immune defense and higher infection risk, which is of particular importance especially in very vulnerable patients, such as preterm and term neonates with an immature immune system.

Congenital infections and especially neonatal sepsis are a frequent cause for morbidity and mortality of newborns [13,14,15,16]. Vertical bacteria transmission from mother to child results in early-onset infections, defined by an onset within 72 h postnatum [16]. The symptoms of a systemic inflammation by newborns are unspecific [13], and the diagnosis is challenging. Isolation of bacteria from blood cultures of neonates is difficult due to the relatively high blood volumes and long incubation times required, and by the initially low bacteria counts in the majority of affected neonates [13,14]. The cytokine interleukin 6 (IL-6) and the acute-phase reactant C-reactive protein (CRP) are important diagnostic markers for early (IL-6) or later (CRP) phases of inflammation [15]. Despite their usefullness, the current clinical algorithm does not provide a satisfying diagnostic specificity in neonates [14], implying that additional biomarkers are needed to facilitate the correct diagnosis and to avoid unnecessary drug administrations. No consented diagnostic and therapeutic guidelines for newborn sepsis have been established. The current therapeutic strategy advocates an early application of an empiric antibiotic treatment if an infection is just suspected [17]. Some antibiotics are applied in terms of an off-label use due to a lack of intervention studies in newborns, raising concerns about their safety [17]. Despite the supportive and antibiotic treatments, congenital infections may still cause long-term complications such as brain damage and neurological sequelae [18]. 

From animal and clinical studies with adult patients, it is well established that inflammatory cytokines disturb the trace element homeostasis [9]. Furthermore, the Cu/Zn ratio is altered in certain diseases [19,20,21,22,23]. Therefore, we postulated that infections disrupt homeostasis of the trace elements Cu and Zn in neonates.

## 2. Materials and Methods 

### 2.1. Study Design

The design of this prospective observational case-control study has been described before in the context of the effects of congenital infections on the selenium (Se) status [24]. Briefly, this explorative study was conducted on the neonatology wards of the Charité-Universitätsmedizin Berlin from February 2013 until April 2014, after clearance with the local ethics committee (approval no.: EA2/092/12). Informed written consent was obtained for each neonate enrolled in the study from the legal guardian(s) prior to analysis. Relevant clinical data were extracted from the electronic and traditional paper-based medical files. Trace elements were analyzed using residual plasma samples from routine laboratory evaluations ordered by the attending physician. Hereby, one sample was collected from each neonate (control or infection) at birth (day 1). The 2nd sample was obtained 48 h later (day 3) only from infected neonates. 

### 2.2. Study Population

Neonates were screened for fulfilling the inclusion criteria of early-onset infection according to published recommendations and the diagnostic steps taken [25,26,27]. In brief, neonates qualifying for the infection group had to exhibit at least one of the following clinical signs: pneumonia, respiratory distress, tachycardia, bradycardia, fever (>38.5 °C), hypothermia (<36.0 °C), irritability, lethargy, hypotonia, poor feeding, increased frequency of apnea, and/or coagulation disorder in combination with laboratory evidence for an inflammation (IL-6 > 100 ng/L, or CRP > 10 mg/L). In neonates with suspected infection, blood cultures were performed prior to antibiotic treatment. Blood cultures were performed with the BacT/ALERT automated system (Organon Teknika, Eppelheim, Germany) in Pedi-BacT pediatric blood culture bottles capable of detecting anaerobic as well as aerobic bacteria. Neonates with suspected early-onset infection were immediately treated with ampicillin and gentamicin for at least 3 days, and a second blood drawing was conducted 48 h later to determine inflammation markers and gentamicin levels. Two residual plasma samples were, thus, available from each of the neonates with suspected infection (day 1 and day 3), and one from the control neonates at time of birth (day 1). The newborns in the control group showed no laboratory evidence for inflammation (IL-6 < 100 ng/L, and/or CRP < 10 mg/L), and were not receiving antibiotic treatment during the hospital stay. Several infants had to be excluded because of birth before 30 weeks of gestation, birth weight below 1000 g, a diagnosed genetic disease, severe congenital malformation, parenteral supplementation with trace elements, or a missing written consent. Details on the neonates enrolled into this study have been published earlier in relation to analyzing their Se status [24] and are provided below (Table 1). Neonates with suspected early-onset infection are summarized as the “infection group” and are denoted as “infected neonates” in this scientific report. However, it needs to be pointed out that a suspected early-onset infection in neonates cannot as safely be diagnosed as in adults for a number of reasons including unspecific symptoms, a higher variability of symptoms, and a very limited amount of blood to be analyzed by laboratory tests and blood culture.

### 2.3. Trace Elements

Total plasma Cu and Zn concentrations were determined by total reflection X-ray fluorescence (TXRF) as described in [24]. The method was chosen because of the low sample volume requirements, which is necessary particularly for infants, and prospectively because of the short time needed for analysis, which is a prerequisite for considering the technique in routine clinical decision-making. Briefly, 10 µL of plasma samples were diluted with 10 μL of a gallium standard solution (f.c. 550 μg/L, Sigma-Aldrich, Steinheim, Germany) and mixed thoroughly. Duplicates of 8 μL each were applied to ultra clean quartz glass carriers, dried at 37 °C and measured using a TXRF spectrometer (S2 PICOFOX, Bruker nano GmbH, Berlin, Germany) as described in [29]. The inter-assay coefficient of variation (CV) was less than 10% for both Cu and Zn. 

### 2.4. Ceruloplasmin

Western blot analysis was performed for assessment of plasma CP levels. Three Western blots were prepared containing plasma samples from neonates of the control group along with samples from infected neonates. An additional Western blot was prepared with samples from the group of infected neonates that were pre-selected with respect to the CRP levels. Plasma was diluted in ultrapure H_2_O (Biochrom AG, Berlin, Germany) and 4x sample buffer (200 mM Tris-HCl, pH 7.5, 50% glycerin, 4% SDS, 0.04% bromophenol blue, and 125 mM DTT). Plasma proteins were size-fractionated by sodium dodecyl (lauryl) sulfate-polyacrylamide gel electrophoresis (SDS-PAGE) and subsequently transferred onto nitrocellulose membranes by semi-dry blotting (Optitran, Schleicher & Schuell, Dassel, Germany). Antibodies against CP (1:2000 dilution, ab19171, Abcam, Cambridge, UK) were used and bands visualized by chemiluminescence (Western-Bright Substrate Sirius, Biozym Scientific, Oldendorf, Germany) using the Fluor Chem FC2 detection system (Biozym Scientific). Quantification of Western blot signals was achieved by using the Java-based image processing program ImageJ (NIH, Bethesda, MD, USA).

### 2.5. Interleukin 6 and C-reactive Protein 

The IL-6 and CRP values were measured by Labor Berlin, Charité Vivantes GmbH, Germany, by standard laboratory analyses as described earlier [24]. Briefly, IL-6 was measured by an electro-chemical luminescence immunoassay and CRP was determined by a turbidometric assay (COBAS 8000 or COBAS 6000; Roche Diagnostics, Mannheim, Germany). 

### 2.6. Statistical Analysis

Statistical analysis was performed with the Statistical Package for the Social Sciences (SPSS Statistics 21^®^, IBM, Chicago, IL, USA) and GraphPad Prism (GraphPad Software Inc., San Diego, CA, USA). Normal distribution of values was assessed by the Shapiro-Wilk test. A two-tailed T-test for unpaired or paired variables and the bivariate Pearson correlation test were used for normally distributed values. For not-normally distributed variables, the Mann-Whitney-U-test, the Wilcoxon-test, and the Spearman´s correlation test were used. The quantified Western blot signals were analyzed using nonparametric tests (Mann-Whitney-U-Test, the Wilcoxon-Test, and the Spearman’s correlation test). Linear regression analysis was conducted to specify associations of variables. Multiple logistic regression was performed to evaluate the results in consideration of relevant confounders. Odds ratios where calculated to examine the quality of the Cu/Zn ratio as a biomarker. The results were considered as statistically significant when the *p*-value was less than 0.05, and differences are marked as follows: *p* < 0.05 (*), *p* < 0.01 (**), and *p* < 0.001 (***). Parametric data are represented as means ± standard deviation (SD) or medians and interquartile range (IQR); median [IQR].

## 3. Results

### 3.1. Cu and Zn Status at Day of Birth in Control and Infected Newborns

Out of 108 newborns, a total of 72 neonates qualified for analysis. Written informed consent was provided for 44 of the samples, of which 23 fulfilled criteria for the control and 21 for the infection group (Table 1). Newborns in the control group had on average a lower gestational age, and lower birth weight. There were more preterm infants (<37 weeks of gestation) in the non-infection control than in the infection group, and more Caesarian sections in the control group (preterm: 13 in control and 5 in infection group vs. term: 3 in control vs. 6 in infection group). There was a variety of clinical symptoms displayed by the infected newborns (Table 2), whereas the newborns in the control group were not displaying any of these signs in combination with laboratory evidence of an inflammation. None of the neonates in the infection group had a positive blood culture. None of the infected or control children had severe thrombocytopenia or leukocytopenia, or displayed any specific signs for an infection with toxoplasma gondii, rubella virus, cytomegalovirus, or herpes simplex virus 1 or 2 (TORCH). No mother had a history of TORCH infection during pregnancy.

**Figure 1:**
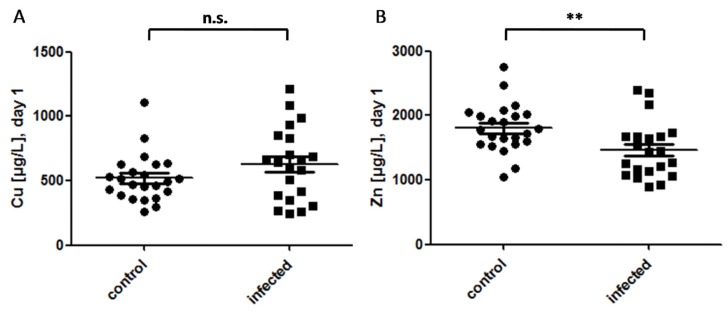
Plasma Cu and Zn in control and infected neonates. (**A**) The Cu concentrations in plasma are not significantly different between the groups of control (*n* = 23) and infected (*n* = 21) neonates on the day of birth (day 1); Mann-Whitney U Test: U = 184; Z = −1.351; *p* = 0.177; (**B**) Infected neonates exhibit significantly lower Zn concentrations as compared to that of control neonates; T-Test (two-sided, unpaired), **: *p* < 0.01.

The median concentration of plasma Cu of all neonates on day 1 was 522.8 [387.1–679.7] μg/L. There was no significant difference between the control and the infection group. The distribution of plasma Cu concentrations was more heterogeneous in the infection group as compared to that of the control group (Figure 1A). Zn concentrations were normally distributed in both groups of newborns, and were higher in the control group as compared to that of the infection group (1804.6 ± 377.0 vs. 1464.8 ± 439.3 µg/L, *p* = 0.009) (Figure 1B). When separating the newborns according to infection and gestational age, Zn levels were significantly lower in the infected term infants as compared to that of the control term infants (1430.3 ± 374.4 vs. 2021.0 ± 488.7 µg/L; *p* = 0.01), but there was no difference in Zn concentration between the infected vs. control preterm neonates.

### 3.2. Cu and CP in Relation to Gestational Age and Infection

**Figure 2:**
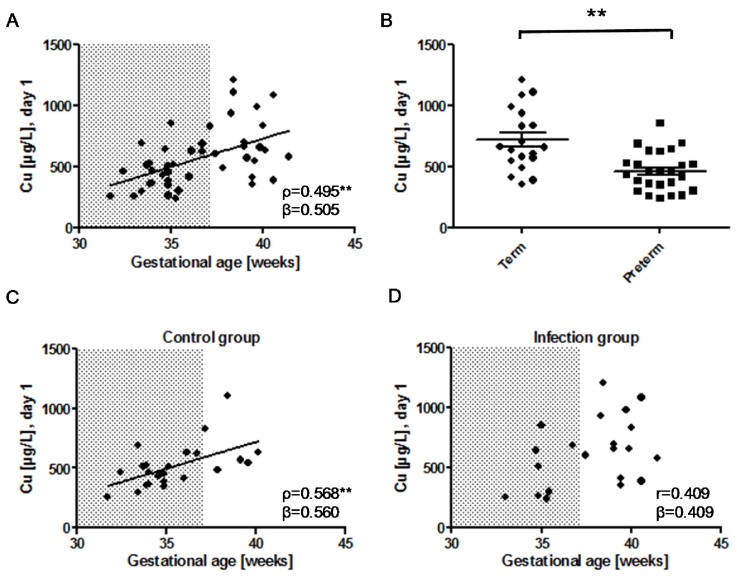
Association of plasma Cu at birth with gestational age. (**A**) In the combined (infection plus control) group, plasma Cu at birth (day 1) correlates positively with gestational age; (**B**) In the combined (infection plus control) group, mean Cu concentration at day 1 is higher in term as compared to that in preterm (<37 gestational weeks) neonates; *n* = 44, T-Test (two-sided, unpaired), *p* = 0.001; (**C**) In the control group, plasma Cu increases with gestational age; (**D**) In infected neonates, there is no significant association of plasma Cu with gestational age. The shaded area (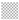) indicates pregnancy length until term, and includes the preterm neonates (<37 weeks of gestation); *ρ*: Spearman’s rank correlation coefficient; r: Pearson correlation coefficient; β: standardised regression coefficient; **: *p* < 0.01.

Plasma Cu concentrations increased with gestational age in the neonates (Figure 2A). When comparing all samples, plasma Cu concentrations were higher in term neonates as compared to that of preterm neonates (715.8 ± 254.0 vs. 461.4 ± 160.5 µg/L; *p* = 0.001) (Figure 2B). There was a significant positive correlation of gestational age and plasma Cu concentrations in the group of control neonates (Figure 2C), but not in the group of infected neonates (Figure 2D). There were no significant differences of the plasma Cu between female and male neonates in the control (491.6 ± 144.1 µg/L (males) vs. 572.0 ± 247.5 µg/L (females); *p* = 0.332) or in the infection group (494.8 ± 195.1 µg/L (males) vs. 681.1 ± 299.7 µg/L (females); *p* = 0.178), when corrected for gestational age and sex.

**Figure 3:**
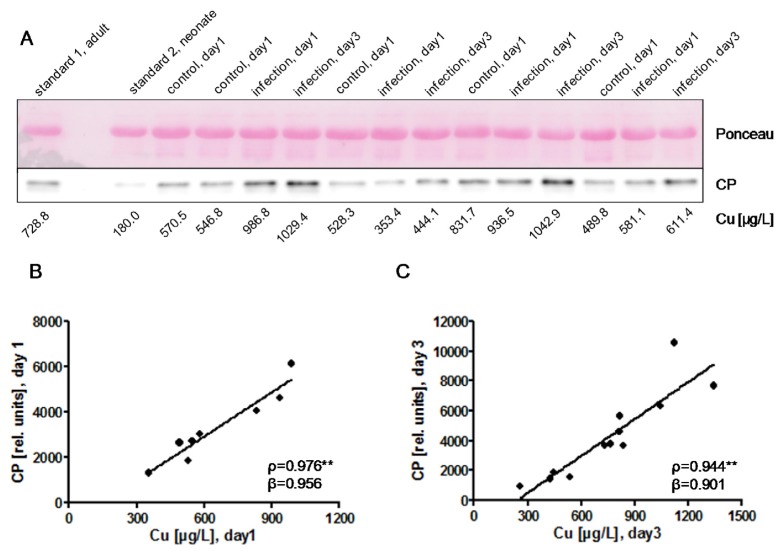
Biomarkers of Cu status in neonates at birth (day 1) and at day 3 after birth. (**A**) Western blot analysis of plasma ceruloplasmin (CP) indicates a single immunoreactive band of 130 kDa. Relatively strong CP signals are observed in plasma samples of infected neonates, especially at day 3. Plasma albumin staining by Ponceau illustrates equal loading of the lanes. The Cu concentrations of the samples are indicated below the Western blot; (**B**) Positive correlation of the CP signal intensities shown in (A) to the plasma Cu concentrations at day of birth (day 1), with some notable exceptions. This association was confirmed by a separate analysis of two further blots (2nd blot; *ρ* = 0.741 *, β = 0.903; 3rd blot; *ρ* = 0.964 **, β = 0.918), implying that both biomarkers are suitable to reflect the Cu status; (**C**) Positive correlation of CP signal intensities in infected neonates to plasma Cu concentrations, determined from a 4th Western blot analysis of plasma samples collected at day 3. *ρ*: Spearman’s rank correlation coefficient; r: Pearson correlation coefficient; β: standardised regression coefficient; **: *p* < 0.01.

Ceruloplasmin (CP) in plasma is an established transport protein for Cu. In order to test whether an early-onset infection affects plasma CP and Cu levels alike, both biomarkers were determined from plasma of infected and control neonates. Western blot analysis detected a single band for CP at the expected size of 130 kDa (Figure 3A). Relative CP units were determined by quantification of the detected signals and showed a significant correlation to the Cu concentrations both at birth (day 1) (Figure 3B) and at day 3 (Figure 3C).

**Figure 4:**
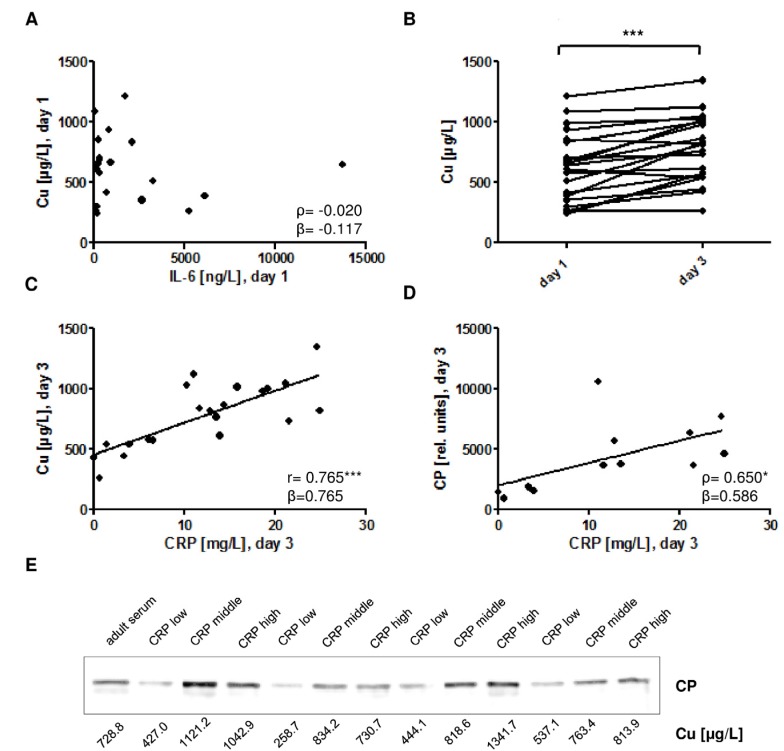
Associations of plasma Cu with established markers of inflammation. (**A**) Plasma Cu concentrations are not associated with the early inflammation marker IL-6 on the day of birth (day 1) in infected neonates; (**B**) Plasma Cu concentrations increase from day 1 to day 3 in infected neonates; *n* = 21, T-Test (two-sided, paired), 627.9 ± 282.5 µg/L vs. 777.1 ± 270.2 µg/L, *p* < 0.001; (**C**) Plasma Cu concentrations correlate positively to the late inflammation marker CRP on day 3 in infected neonates; (**D**) Similarly, CP levels show a positive correlation to CRP in plasma of infected neonates on day 3; (**E**) Newborns were categorized according to severity of infection based on CRP levels (top row; [CRP]: low, < 5 mg/L; moderate, 10–15 mg/L; high, > 20 mg/L). Western blot analysis detected a single immunoreactive CP band and indicated a positive association of CRP levels with CP concentrations in the infected neonates on day 3. Equal protein loading was evaluated by Ponceau staining prior to Western blot analysis. *ρ*: Spearman’s rank correlation coefficient; r: Pearson correlation coefficient; β: standardised regression coefficient; *: *p* < 0.05; ***: *p* < 0.001.

The pro-inflammatory cytokine IL-6 is an early diagnostic marker of congenital infection. In the group of infected newborns, IL-6 was not associated with plasma Cu concentrations at day of birth (day 1) (Figure 4A). Plasma Cu concentrations increased in infected newborns from day 1 to day 3 (Figure 4B). The acute phase protein CRP is determined as late biomarker of neonatal infection. CRP on day 3 was positively associated with plasma Cu concentrations both on day 1 (r = 0.720, *p* < 0.001, β = 0.720) and on day 3 (Figure 4C). A similar positive correlation was detected between plasma CP and CRP concentrations (Figure 4D). The CP detected in plasma of infected newborns ran as a single protein of the expected size, and it was relatively abundant in samples with elevated CRP levels (Figure 4E).

### 3.3. Associations of Zn with Gestational Age, Birth Weight, and Infection

**Figure 5:**
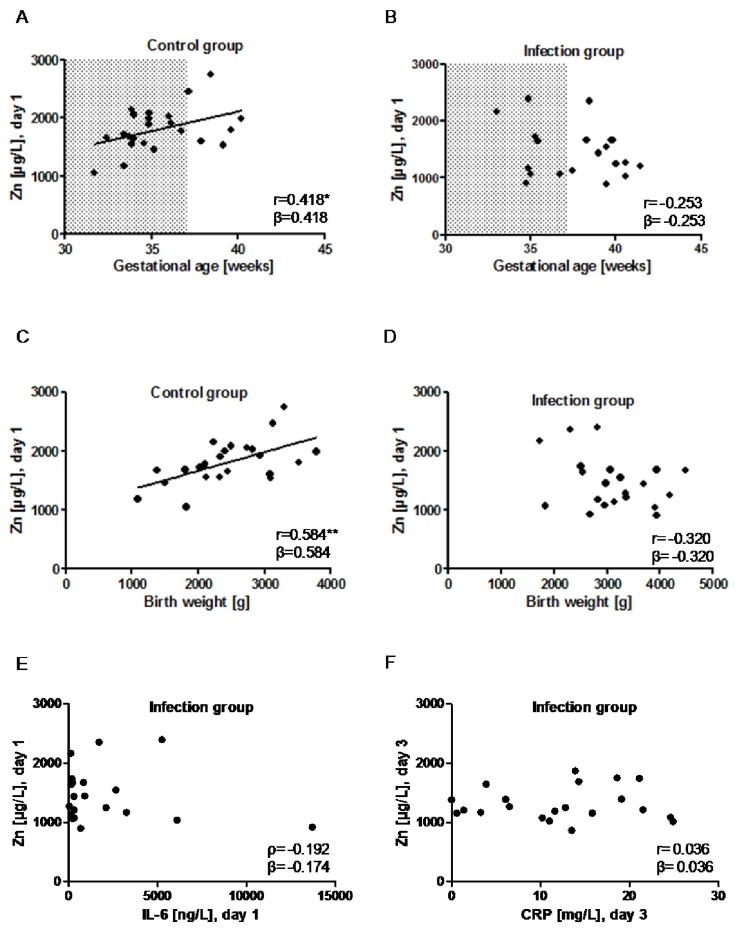
Associations of plasma Zn with gestational age, birth weight, and infection markers. (**A**) There is a positive correlation between plasma Zn and gestational age in the control group; (**B**) In infected neonates, plasma Zn and gestational age are not related; (**C**) Similarly, plasma Zn is positively associated with birth weight in the control group; (**D**) but not in the group of infected neonates; (**E**) There is neither an association of plasma Zn concentrations with the early marker of infection, i.e., IL-6; (**F**) nor with the late marker CRP. The shaded area (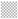) indicates the preterm neonates (<37 weeks of gestation); *ρ*: Spearman’s rank correlation coefficient; r: Pearson correlation coefficient; β: standardised regression coefficient; *: *p* < 0.05; **: *p* < 0.01.

Plasma Zn concentrations correlated positively to gestational age in control neonates (Figure 5A). In comparison, no significant correlation was observed in the group of infected neonates (Figure 5B). Similarly, plasma Zn concentrations were positively correlated with birth weight in the group of controls (Figure 5C) but not in infected neonates (Figure 5D). There were no significant differences of the plasma Zn concentrations between female and male neonates either in the control group (1739.4 ± 316.2 µg/L (males) vs. 1926.8 ± 469.7 µg/L (females); *p* = 0.266) or in the infection group (1308.9 ± 234.3 µg/L (males) vs. 1527.2 ± 491.4 µg/L (females); *p* = 0.316). The diagnostic markers of inflammation determined in the group of infected neonates, i.e., IL-6 on day 1 and CRP on day 3, were not associated with the plasma Zn concentrations at day 1 (Figure 5E) or at day 3 (Figure 5F).

### 3.4. Cu/Zn Ratio

**Figure 6:**
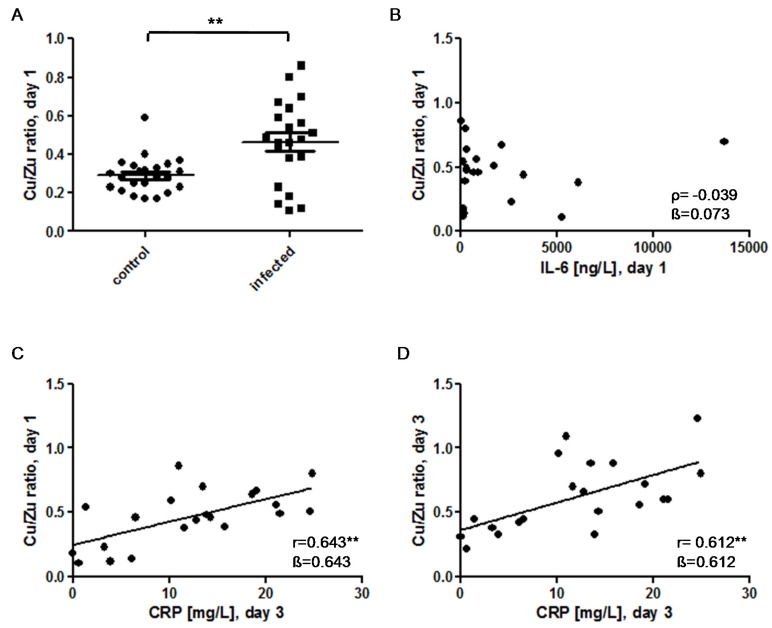
The Cu/Zn ratio as a diagnostic marker for early-onset congenital infections. (**A**) The Cu/Zn ratio is significantly elevated in the infection group (0.28 [0.23–0.34] vs. 0.48 [0.30–0.61]; U = 118; Z = −2.902; *p* = 0.004); (**B**) There is no correlation between the Cu/Zn ratio and IL-6 on the day of birth (day 1) in infected infants; (**C**) The Cu/Zn ratio on day 1 correlates positively with the CRP-values on day 3 after birth in the infection group; (**D**) Similarly, the Cu/Zn ratio on day 3 correlates positively with the CRP levels on day 3 in the infected neonates. *ρ*: Spearman’s rank correlation coefficient; r: Pearson correlation coefficient; β: standardised regression coefficient; **: *p* < 0.01.

In our quest for a more robust parameter, which may complement the current diagnostic tools for suspected early-onset infections, the Cu/Zn ratios were calculated. This ratio was significantly higher in the infection group as compared to that of the control group of neonates (Figure 6A). In infected neonates, there was no association of the Cu/Zn ratio with the IL-6 levels on day 1 (Figure 6B). However, the CRP levels determined at day 3 in infected neonates showed a positive correlation to both the Cu/Zn ratios at the day of birth (day 1) (Figure 6C) and at day 3 (Figure 6D). Furthermore, the plasma Cu/Zn ratio was correlated with gestational age in the control group (*ρ* = 0.417*, *p* = 0.048) but not in the infection group (r = 0.332, *p* = 0.141). 

To verify the reliability of the Cu/Zn ratio as a biomarker for early-onset infection considering the different distribution of gestational age between the groups, we conducted a multinomial logistic regression with gestational age as a covariate. The Odds Ratio for the Cu/Zn ratio (day 1) was 9.067 (95% confidence interval 2.306–35.650), which indicates a high Cu/Zn ratio (>50th-percentile) has higher odds for infection than those in the <50th-percentile-group (Table 3). 

## 4. Discussion

In this study, we evaluate the infection-related differences in plasma Cu and Zn concentrations in preterm and term neonates. Our results indicate that both trace elements increase in plasma with gestational age in control newborns, and that this correlation is lost in infection. Infected neonates show relatively low plasma Zn concentrations at birth, and develop elevated plasma Cu concentrations during infection. As the plasma concentrations of these two trace elements are regulated in opposite directions by infection, we speculated that the Cu/Zn ratio may provide a more robust marker of early-onset infection than either value alone. Our study indicates that the Cu/Zn ratio correlates to the CRP levels determined at day 3 as the established biomarker of infection. Interestingly, this correlation is already shown at day 1. It remains to be tested if it could be of value for the diagnosis of early-onset infections. The Cu/Zn ratio at day 1 may already reflect the severity and predict the potential course of the infection, which then becomes detected later by the elevated CRP levels determined at day 3. It may, thus, constitute a helpful early prognostic biomarker of early-onset infection in term and preterm neonates.

There is evidence that preterm neonates are at especially high risk for Cu and Zn deficiency [4,30]. This notion is supported by previous studies reporting that the maternal Cu levels rise with the length of gestation [31]. Consistent with former findings in neonates [32], the Cu concentrations of the infants in our study were lower, and Zn was higher than in adults (adult reference intervals according to [11]; Cu; 10–22 µM, i.e., 635.5–1398.0 μg/L, and Zn; 12–18 µM, i.e., 784.6–1176.8 µg/L). Our data are in line with previous studies in young children, especially with respect to an increase in plasma Cu levels upon infection [33]. Notably, also in preterm and term infants, a tight correlation of plasma Cu concentrations and plasma CP is reported [34]. These data along with our findings support the concept that plasma CP may serve as a surrogate marker of plasma Cu concentrations in children. This relation offers the option for bed-side testing of the Cu status by immunological assay procedures, as recently demonstrated by using quantum dots for fast CP quantification [35]. Unfortunately, no reliable protein biomarker of plasma Zn status is yet at hand, which would enable a fast multiplex bed-side quantification of the Cu/Zn ratio via these surrogate protein biomarkers that can be detected by point-of-care technologies.

Importantly, the inverse regulation of plasma Cu and Zn is a well-established characteristic of infections, and the Cu/Zn ratio has been proven of diagnostic value in a number of human disorders, including pediatric infectious diseases, such as giardiasis or amebiasis [20] and tuberculosis [23,36]. Furthermore, the diagnostic value of the Cu/Zn ratio as a disease marker was also shown in autism, attention-deficit hyperactivity disorder, hypertension, inflammatory, as well as neoplastic diseases [33,37,38,39]. The quotient was also described as a potential biomarker of inflammation and nutritional status as well as a mortality predictor in elderly people [40], and as a variable correlating with inflammation, disrupted immune system, and an increased oxidative stress in peritoneal dialysis [41]. Our data indicate that the Cu/Zn ratio may also be of diagnostic value in neonates with suspected infection, as it was associated with severity of inflammation at an early time point while being independent of gestational age. 

### 4.1. Early-Onset Congenital Infections as Disruptors of the Trace Element Homeostasis

We found significantly lower plasma Zn concentrations in infected neonates compared with that of the control group, which is congruent with former findings in children, adults, and animals suffering from an acute inflammation and/or critical illness [3,7,10,11,42]. However, Zn levels were not associated with the early inflammation marker IL-6 or with the late acute phase reactant CRP. This lack of stringent interrelation is in line with studies in adults, where there are only marginal correlations of serum Zn with markers of inflammation [43]. This may indicate that low plasma Zn may not reflect the severity of the inflammation, i.e., it may not be directly regulated by the cytokines released in response to infection. However, in other studies, a respective correlation of plasma Zn with inflammation markers in critically ill children and adults has been reported [11,44]. The lower Zn levels in our infected neonates may, thus, not necessarily be the consequence of the inflammation, but potentially a risk factor for infection [8]. However, this hypothesis needs to be tested in other clinical trials, as our analysis is an observational study and not designed to identify causal relationships. 

The Cu levels increased with gestational age. In general, the fetal hepatic tissue does not efficiently support an incorporation of Cu into the CP apoenzyme [32]. Thus, the increasing Cu levels with age may reflect the functional maturation of the liver. Due to ethical reasons, only residual plasma samples were available from infected neonates at day 3 but not from control neonates. Such an analysis would shed light on the relative importance of age and infection for the rising Cu concentrations observed in the study. Moreover, the diagnosis of infection in the newborns was based on clinical symptoms in combination with laboratory evidence for an inflammation, and not on positive blood cultures or additional laboratory analysis, which constitutes a general shortcoming of our study.

### 4.2. The Cu/Zn Ratio as a Potential Biomarker of Early-Onset Congenital Infections

The concentrations of plasma Cu or Zn considered separately did not qualify as useful biomarkers of early-onset infection. The correlation of Cu on day 1 with CRP on day 3 seems to impart a predictive value to the trace element, and the association on day 3 implicates its potential as a clinical marker of disease course. However, Cu levels were not significantly different between control and infected subjects at birth. The strong positive association of CP and CRP on day 3 suggests that CP is related to the severity of infection. CP is described as a positive acute phase reactant in adults [4,9]. However, it decreases with time during increasing severity of the inflammation in adults [11,45], suggesting that it does not steadily correlate to infection severity. Zn levels were significantly lower in infected neonates compared with that of the control group at birth. However, Zn neither correlated with IL-6 on day 1, nor with CRP on day 3. Considering these interactions, Zn alone does not seem to quality as an appropriate infection marker in neonates.

Nevertheless, the Cu/Zn ratio appears to provide additional information on the possible infection of newborns. This notion needs to be tested in prospective trials with a sufficient number of neonates, as both the infection and control groups were relatively small in our pilot study.

## 5. Conclusions 

Infection as well as inflammation can affect the trace element homeostasis in newborns. Infected neonates may develop increased plasma Cu concentrations and display a relative plasma Zn deficit. The Cu/Zn ratio may thus constitute a useful biomarker of early-onset infection in neonates with some relation to the clinical course.

## Figures and Tables

**Table 1:** Characteristics of the study population, as previously described [24].

	Control Group	Infection Group	*p*-Value (Two-Sided)
Participants	*n* = 23	*n* = 21	-
Gestation age [weeks]	34.9 [33.9–37.1]	38.4 [35.1–39.8]	0.003
Term infants	*n* = 6 (26%)	*n* = 13 (62%)	
Preterm infants (<37 gestation weeks)	*n* = 17 (74%)	*n* = 8 (38%)	
Birth Weight [g]	2452.3 ± 694.1	3119.7 ± 733.7	0.003
Vaginal birth	*n* = 7 (30%)	*n* = 10 (48%)	-
Twins	*n* = 7 (30%)	*n* = 1 (5%)	-
Apgar 1 min *	9.0 [6.0–9.0]	8.0 [4.5–9.0]	0.505
Apgar 5 min *	9.0 [8.0–10.0]	9.0 [7.5–10.0]	0.951
Cord arterial pH	7.25 [7.2–7.29]	7.23 [7.19–7.3]	0.655
pH on admission ^#^	7.33 [7.27–7.38]	7.33 [7.26–7.37]	0.673
Base excess on admission ^##^	−2.15 [(−3.75)–(−0.90)]	−2.8 [(−5.32)–(−1.28)]	0.283
IL-6 [pg/mL], day 1	5.8 [4.2–17.0]	498.5 [203.6–2523.0]	<0.001

* The Apgar score is used to characterize the condition of neonates after birth [28]. The Apgar score evaluates the condition of the neonate 1, 5, and 10 min after birth and guides subsequent interventions. Furthermore, the 5 min Apgar score has been used to predict neurological long-term-outcome. Each of five easily identifiable characteristics–respiration, heart rate, skin color, reflex irritability, and muscle tone—is assessed and assigned a value of 0 to 2. ^#^ The blood pH was measured in each neonate immediately after admission to the neonatal ward. ^##^ The base excess refers to an excess or deficit in the amount of base in the blood. A negative base excess reflects an acid/base disturbance caused by the accumulation of lactate acid because of anaerobic glycolysis. IL stands for interleukin.

**Table 2:** Clinical characteristics of the neonates with suspected early-onset infection (*n* = 21). IL stands for interleukin; IRQ stands for interquartile range; CRP stands for C-reactive protein; SD stands for standard deviation.

Clinical Characteristics	
Pneumonia/Respiratory Distress	*n* = 19
Tachycardia/bradycardia	*n* = 6
Fever/hypothermia	*n* = 2
Irritability/lethargy	*n* = 3
Coagulation disorder	*n* = 0
IL-6 [ng/L], day 1 (median [IQR])	498.5 [203.6–2523.0]
CRP [mg/L], day 3, (mean ± SD)	12.1 ± 7.8
Antibiotic treatment [days] (median [IQR])	5.0 [3.0–5.0]

**Table 3:** Association of Cu/Zn ratio and infection adjusted for gestational age by multiple logistic regression analysis. CI: confidence interval.

	Significance	Odds Ratio	95% CI
Gestational age	0.079	1.310	0.969–1.770
Cu/Zn ratio	0.034	164.224	1.457–18,506.693
